# The Impact of Scars After DIEP-Flap Breast Reconstruction on Satisfaction and HR-QoL: A Cross-Sectional Study Comparing BREAST-Q Scores

**DOI:** 10.1007/s00266-024-04272-y

**Published:** 2024-09-03

**Authors:** Kristel E. Everaars, Erik H. de Laat, Danny A. Young-Afat, Esther P. M. Tjin, Dietmar J. O. Ulrich

**Affiliations:** 1 Department of Plastic Surgery, Radboudumc, Geert Grooteplein-Zuid 10, 6500 HB Nijmegen, The Netherlands; 2 Research Center Healthy and Sustainable Living, Research group Innovation in Healthcare Processes in Pharmacology, University of Applied Sciences Utrecht, Heidelberglaan 7, 3584 CS Utrecht, The Netherlands; 3 Department of Plastic, Reconstructive and Hand Surgery, Amsterdam University Medical Center, Boelelaan 1118, 1081 HZ Amsterdam, The Netherlands

**Keywords:** Breast reconstruction, DIEP flap, Scar, POSAS, BREAST-Q, Patient education

## Abstract

**Background:**

Although deep inferior epigastric perforator (DIEP) flap breast reconstruction is the most widely used technique for autologous breast reconstruction, this technique leads to large scars in visible areas on breast and abdomen. So far, limited studies have thoroughly addressed the impact of breast and abdominal scars on satisfaction and Health-related Quality of Life (HR-QoL).

**Objectives:**

This research aimed to determine whether women with no/minor scar symptoms after undergoing DIEP-flap breast reconstruction differ in satisfaction and perceived HR-QoL from women with symptomatic scars.

**Materials and Methods:**

In this cross-sectional survey study, women who had previously undergone DIEP-flap breast reconstruction completed an online survey. Patient-reported scar quality was assessed with the Patient and Observer Scar Assessment Scale (POSAS), and satisfaction and HR-QoL with BREAST-Q. Independent-samples t-tests were conducted to compare BREAST-Q scores between women with no/minor scar symptoms (POSAS overall opinion score 1–3) and women with symptomatic scars (POSAS overall opinion score 4–10).

**Results:**

A total of 248 women completed the survey. Women with scar symptoms had significantly worse BREAST-Q scores on ‘Satisfaction with breasts,’ ‘Physical well-being,’ ‘Psychosocial well-being’ and, ‘Sexual well-being’ compared to women with no/minor scar symptoms (*p* ≤ 0.001).

**Conclusion:**

After DIEP-flap breast reconstructions, women with symptomatic breast and abdominal scars had a clinically relevant and statistically significant lower degree of satisfaction and HR-QoL compared to women who had no/minor scar symptoms. We therefore recommend to explicitly and repeatedly address inevitability of visible scars after DIEP-flap breast reconstruction, aiming to improve preoperative patient selection and post-operative expectation management.

**Level of Evidence IV:**

This journal requires that authors assign a level of evidence to each article. For a full description of these Evidence-Based Medicine ratings, please refer to the Table of Contents or the online Instructions to Authors www.springer.com/00266.

**Supplementary Information:**

The online version contains supplementary material available at 10.1007/s00266-024-04272-y.

## Introduction

Successful breast reconstruction following a mastectomy can greatly improve patient’s perceived health-related quality of life (HR-QoL) compared to women who do not undergo breast reconstruction [1]. Recent systematic reviews show that women undergoing autologous breast reconstructions have superior patient-reported outcomes over those undergoing implant-based reconstructions [2–4]. The deep inferior epigastric perforator (DIEP) flap has become the most widely used technique for autologous breast reconstruction [4, 5]. Furthermore, compared to other autologous reconstruction techniques, patients undergoing DIEP-flap reconstruction have the highest level of satisfaction [4, 6].

Nevertheless, DIEP-flap breast reconstruction also leads to large scars in the breast and abdominal region. In previous research [7], we explored patient-reported breast and abdominal scar quality after DIEP-flap breast reconstruction in detail with the validated scar assessment scale ‘Patient and Observer Scar Assessment Scale’ (POSAS). This study revealed that, after DIEP-flap breast reconstruction, most women experienced clinically relevant scar symptoms on at least one scar characteristic of their breast and/or abdominal scar (especially color, stiffness, thickness, and irregularity). Furthermore, abdominal scars were appraised significantly worse than breast scars.

These insights from our previous research indicated that patient-reported scar quality is an essential outcome after DIEP-flap breast reconstruction. Moreover, other studies show that scars resulting from (various types of) breast reconstruction might potentially influence overall satisfaction and Health-related Quality of Life (HR-QoL) [8–11]. However, these insights are derived from studies using qualitative methods [10, 11], considering solely breast scars and not the donor site scars [9], or do not use validated measurement instruments [8]. This compelled us to conduct further research on the influence of both breast and abdominal scarring after DIEP-flap breast reconstruction on patient-reported satisfaction and HR-QoL.

Therefore, we performed a survey study aimed at evaluating the impact of patient-reported breast and abdominal scarring (i.e., assessed with the POSAS) on satisfaction and HR-QoL (i.e., assessed with the BREAST-Q). We were specifically interested in whether there are differences between women with no/minor scar symptoms and those with more pronounced scar symptoms, providing clinically relevant knowledge that may then be directly applied in daily practice to enhance expectation management.

## Methods

### Design

This explorative cross-sectional study is the second part of an overarching study, in which women after DIEP-flap breast reconstruction were invited to complete an online survey on breast and abdominal scarring [7]. Where in the first study patient-reported breast and abdominal scar quality after DIEP-flap breast reconstruction was explored in detail, the current study focusses on the impact of the patient-reported scar quality on satisfaction and HR-QoL. This study was reported according to the reporting items stated in the Strengthening the Reporting of Observational Studies in Epidemiology (STROBE) [12]***.***

### Setting

The online survey was distributed between February 17 and May 5, 2019, among a diverse population of women who had undergone a DIEP-flap breast reconstruction. Women from two hospitals (Radboud University Medical Center and Maastricht University Medical Center, the Netherlands) were invited by e-mail to participate. This included 516 women who had previously given their consent to participate in scientific research. In addition, an open invitation was sent through social media allowing women from other hospitals in the Netherlands to participate, aiming for a diverse sample [7].

### Participants

Women were eligible to participate if they had undergone a DIEP-flap breast reconstruction at least 6 weeks ago, with a maximum of 10 years ago, after a prophylactic or curative, unilateral or bilateral, total or skin-sparing mastectomy. Women were asked to participate regardless of the presence or absence of scar symptoms, and irrespective of whether they had (undergone) additional therapy for their scar(s) [7].

### Outcomes

The survey consisted of items regarding patient and surgical characteristics, patient-reported scar quality, satisfaction and, HR-QoL. Patient-reported breast and abdominal scar quality was assessed with the patient assessment scale of the validated Dutch version of POSAS 2.0, with higher scores indicating more scar symptoms [13, 14]. The POSAS was completed twice within the same session; once for the patient-reported breast scar quality and once for the patient-reported abdominal scar quality. The patient-reported scar quality was measured with the POSAS *Overall opinion.*

Satisfaction and HR-QoL were evaluated with scales from the breast reconstruction module of the BREAST-Q (version 2. 0) [15], a validated instrument to quantify the patient’s perception after breast reconstructive surgery [16]. For each BREAST-Q scale, a separate score was calculated, based on the official BREAST-Q scoring manual. As described in the manual, scores were converted from a Rasch-based score to a 0–100 score, with higher scores reflecting higher patient satisfaction or better HR-QoL [17]. Satisfaction was assessed using two scales of the BREAST-Q: *Satisfaction with breast* and *Satisfaction with abdomen*. HR-QoL was assessed with the scales: *Physical well-being* (breast and abdomen), *Psychosocial well-being* and *Sexual well-being*.

All BREAST-Q scales had to be filled out completely, except for the scale ‘Sexual well-being.’ Women were able to answer ‘not applicable’ when uncomfortable answering these questions or if they did not feel that the questions applied to them. Based on BREAST-Q scoring instructions, if less than 50% of the scale’s item were missing, the mean was inserted for the missing items. If missing data were more than 50% of the scale’s item, it was handled as missing, as described in the official manual [17].

### Ethics

This study was performed in line with the principles of the Declaration of Helsinki. Approval was granted by the Medical Ethics Committee of the Radboudumc Nijmegen, The Netherlands (registration number 2018-4916). All participants gave explicit consent for study participation.

### Statistical Analysis

The patient-reported scar outcomes were analyzed using descriptive statistics. Due to the absence of clinically relevant cut off points, two groups were created based on previous literature [7, 18]. The first group representing women after DIEP-flap breast reconstruction reporting no or minor differences compared to the normal skin of their breast or abdominal scar (POSAS scores 1–3), indicating no/minor scar symptoms, the second group representing major differences with normal skin, indicating scar symptoms (POSAS scores 4–10) [7, 18]. Independent-samples t-tests were conducted to compare BREAST-Q scores for women with POSAS scores 1–3 (i.e., no/minor scar symptoms) and for women with POSAS scores 4–10 (i.e., scar symptoms). The mean *breast* POSAS overall opinion score was compared with the BREAST-Q scales ‘Satisfaction with breasts,’ ‘Physical well-being: chest,’ ‘Psychosocial well-being’ and, ‘Sexual well-being.’ The mean *abdominal* POSAS overall opinion score was compared with ‘Physical well-being: abdomen’ and, ‘Sexual well-being.’

To explain the proportion of variance in the BREAST-Q scores by POSAS scores, the Eta squared was calculated: Eta squared = *t*^2^/*t*^2 ^+ (*N*1 + *N*2–2), were 0.01 = small effect, 0.06 = moderate effect and, 0.14 =  large effect [19]. A Bonferroni adjustment was applied to reduce the chance of a Type 1 error because we performed multiple statistical tests, and results were considered significant at *p *≤ 0.01 [19]. A sensitivity analysis to assess the impact of time was used to compare the POSAS total scores for scar maturation to visualize the impact of scar maturation on satisfaction and well-being measured with the BREAST-Q scores. Tentatively, it has been chosen to conduct a sensitivity analysis on scars younger than 1 year and scars older than 1 year, as well as scars younger than 1.5 years and scars older than 1.5 years. These cutoff points have been selected due to the average maturation period of surgical scars [20].

Complete case analysis was used in case of missing data. SPSS Statistics, version 25 (IBM Corp., Armonk, N.Y.) was used for the analyses.

## Results

A total of 252 women completed the questionnaires; four women retrospectively did not meet the inclusion criteria and were therefore excluded from analysis. Participants had a mean age of 52 years (27–74 years). Mean time from breast reconstruction to questionnaire completion was 31.4 months ago (1.3–123.2 months). Most women (75%) had a history of breast cancer (See Table 1: patient characteristics). The patient characteristics were also divided based on the presence or absence of scar symptoms and were generally equal distributed (See Digital Supplement 1: Comparison of Patient Characteristics Based on the Presence or Absence of Scar Complaints).

**Table 1:** Patient characteristics

*N* = 248	Mean ± SD
Age (year)	51.8 ± 9.0
Years since DIEP	2.6 ± 1.9
Months since DIEP	31.4 ± 22.3
BMI (kg/m^2^)	27.1 ± 3.5

	*N*	%
Bra cup size		
< *D*	150	60
≥ *D*	98	40
Time of reconstruction^a^		
Direct	68	28
Indirect	179	72
Laterality		
Unilateral	140	56
Bilateral	108	44
History of breast cancer		
Breast cancer history	187	75
No breast cancer history	61	25
Irradiation^b^		
Yes	99	40
No	149	60
Self-reported complications^c^		
Breast	105	42
Abdomen	105	42
Self-reported current symptoms		
Breast	110	44
Abdomen	94	38

^a^*n* = 247, missing due to incorrect data entry for date.

^b^43 participants had a bilateral prophylactic mastectomy, no radiation was indicated.

^c^Some of the women (*n* = 58) had both breast and abdominal complications, however, the number of complications happened to be equally high.

Statistically significant differences were found on all BREAST-Q scales comparing patient-reported scar quality for women reporting no/minor scar symptoms (POSAS 1-3) and women reporting scar symptoms (POSAS 4-10) (*p *≤ 0.001) for both breast and abdominal scars (See Table 2. Patient-reported outcomes BREAST-Q and POSAS and Table 3. BREAST-Q scores). Considering the mean difference of the 6 different BREAST-Q scales, the mean difference was largest for the BREAST-Q scale ‘Satisfaction with breasts’. For ‘Satisfaction with breasts’ there was a significant difference in scores between women with no/minor breast scar symptoms (*M* = 74.5 ± 16.0) and women with breasts scar symptoms (*M* = 57.3 ± 15.5, *p *= <0.001). A large proportion of the variance (23%) could be explained by the patient-reported breast scar quality, with a mean difference of 17.2. Considering the mean differences in BREAST-Q scores between women with no/minor breast scar symptoms and those with breast scar symptoms, the range was from 13.3 to 17.2 (*p* ≤ 0.001). Similarly, taking into consideration the mean difference in BREAST-Q scores between women with no/minor abdominal scar symptoms and those with scar abdominal symptoms, the range was from 11 to 13.2 (*p* ≤ 0.001). Overall, the effect sizes were moderate, which implies that the variance of BREAST-Q scores was moderately explained by the patient-reported scar quality (See Table 3. BREAST-Q scores).

**Table 2:** Patient-reported outcomes BREAST-Q and POSAS

BREAST-Q	Mean ± SD
Satisfaction with breast	66.1 ± 17.9
Physical well-being breast	72.7 ± 22.0
Physical well-being abdomen	61.2 ± 20.3
Psychosocial well-being	71.5 ± 19.7
Sexual well-being^a^	55.0 ± 22.2

POSAS	
Total score breast	22.6 ± 12.5
Total score abdomen	25.9 ± 13.4
Overall score breast	4.2 ± 2.4
Overall score abdomen	5.0 ± 2.6

^a^77.8% (*n* = 193) of the 248 women completed the sexual well-being scale completely, in 88.3% (219 of the 248) of the women less than 50% was missing, here the mean could be inserted for the missing items

**Table 3:** BREAST-Q scores: comparing no/minor scar symptoms versus scar symptoms

	No/minor scar symptoms POSAS 1-3 Mean ± SD	(major) scar symptoms POSAS ≥ 4 Mean ± SD	Mean difference	Sig. 2-tailed	95% CI lower	95% CI upper	Eta squared *η*^2^ (% variance)	Normative values^e^	Cut off points^f^
**BREAST**									
Satisfaction^a^	74.5 ± 16.0	57.3 ± 15.5	17.2	< 0.001	13.3	21.1	0.23 (23%)	58 ± 18	73
Physical well-being^a^	79.9 ± 18.7	65.1 ± 22.7	14.8	< 0.001	9.5	20.0	0.11 (11%)	93 ± 18	78
Psychosocial well-being^a^	78.3 ± 18.2	64.3 ± 18.8	14.0	< 0.001	9.3	18.6	0.13 (13%)	71 ± 18	78
Sexual well-being^b^	61.8 ± 20.5	48.5 ± 21.9	13.3	< 0.001	7.6	18.9	0.09 (9%)	56 ± 18	60
**ABDOMINAL**									
Physical well-being^c^	69.7 ± 17.9	56.5 ± 20.0	13.2	< 0.001	8.3	18.1	0.10 (10%)	78 ± 20	75
Sexual well-being^d^	62.2 ± 24.9	51.2 ± 19.7	11.0	0.001	4.4	17.5	0.05 (5%)	56 ± 18	60

^a^*n* = 248:128 women with no/minor scar symptoms versus 120 women with scar symptoms

^b^*n* = 219: 106 women with no/minor scar symptoms versus 113 with scar symptoms, sexual well-being is administered once in the survey, but analyzed twice: once for the POSAS breast scars, once for the POSAS abdominal scars

^c^*n* = 248: 88 women with no/minor scar symptoms versus 160 with scar symptoms

^d^*n* = 219:75 women with no/minor scar symptoms versus 144 with scar symptoms

^e^Normative values based on 1201 women with or without cancer [16]

^f^Recently used cutoff points for autologous reconstruction by Shammas and colleagues [10]

The sensitivity analysis, which compared the POSAS *total* scores for breast and abdominal scars for more mature with unmature scars showed similar trends in BREAST-Q scores (Digital Supplement 2).

The BREAST-Q scale ‘Satisfaction with abdomen’ consists of stand-alone items, therefore no total score can be derived from the data. A relatively large proportion of women was dissatisfied with the abdominal scar, ranging from somewhat dissatisfied (27.8%) to very dissatisfied (12.5%) (Table 4. Descriptive BREAST-Q scores satisfaction abdomen).

**Table 4:** Descriptive BREAST-Q scores satisfaction abdomen

	Very dissatisfied (%)	Somewhat dissatisfied (%)	Somewhat satisfied (%)	Very satisfied (%)
Satisfaction abdomen unclothed	11.7	20.6	40.3	27.4
Position of navel	2	11.7	32.3	54.0
How abdominal scars look	12.5	27.8	40.7	19.0

## Discussion

In the setting of breast reconstruction surgery, patient satisfaction and HR-QoL are important outcome variables when evaluating surgical success. Our results show that both patient satisfaction and HR-QoL was significantly lower in patients with more scar-related symptoms of the breast and abdominal scars, which underlines that patient-reported scar quality is an important outcome after DIEP-flap breast reconstruction.

A moderate to large proportion of the variation of the satisfaction and HR-QoL, as measured with the BREAST-Q, can be assigned to the patient-reported scar quality. The impact of breast scars, measured with the POSAS overall opinion, is largest on BREAST-Q ‘Satisfaction with breast.’ Breast scars explain 23% of the variation of satisfaction with breasts after DIEP-flap breast reconstruction. Unfortunately, since no total score of the BREAST-Q ‘Satisfaction with abdomen’ scale can be derived, the impact of scarring on satisfaction with abdomen could not be determined. However, based on the descriptive BREAST-Q data for ‘Satisfaction with abdomen,’ we see that a large proportion of women are dissatisfied with the abdominal scar. Apart from the impact of scarring on patient satisfaction, the current study confirms that women with a positive self-reported scar appraisal have a better physical, psychosocial and sexual well-being after DIEP-flap breast reconstruction. The relationship between satisfaction and HR-QoL after DIEP-flap breast reconstruction and scarring of the breast [9, 10, 21] and abdomen [8, 10, 21] was indicated before in previous studies, of which the impact of abdominal scars was exclusively explored qualitatively.

The mean differences observed in the BREAST-Q scales ranged from 11 to 17.2. These differences are substantial, exceeding the recommended Minimal Important Differences for the BREAST-Q scales in clinical research (3 or 4 depending of the scale) [22], indicating both statistical and clinical significance.

Furthermore, women reporting no/minor scar symptoms were more satisfied and had a better psychosocial and sexual well-being BREAST-Q score compared to normative BREAST-Q values. Women with more scar symptoms had worse BREAST-Q scores on all scales, in comparison to normative values (See Table 3. BREAST-Q scores) [16]. Moreover, women with scar symptoms had low BREAST-Q scores on all scales when compared to recently used cut off points [10]. This further highlights the importance of paying attention to scars before and after surgery.

## Clinical Implications

There are many known factors that may affect satisfaction after breast reconstruction such as age, complications [23] psychosocial aspects [9], smoking [24], asymmetry, cup size, and lack of sensation [10]. HR-QoL may also be affected by the type of oncological treatment received, the presence of lymphedema [24], persistent pain, weakness or numbness [10]. It is important to note that there may be a discrepancy between patient’s and professional’s opinions regarding what is considered “acceptable” scarring. Additionally, professionals may prioritize the shape and symmetry of the reconstructed breast over the visibility of the scar [25]. This discrepancy could be explained by the process of coping with physical changes such as scarring, which can serve as an emotional reminder of overcoming breast cancer [26].

Considering scarring as an important risk factor for impaired satisfaction and HR-QoL, is an important implication for future research and clinical practice. For clinical practice, we therefore recommend to always clearly emphasize the inevitability of scars after DIEP-flap breast reconstruction during shared-decision making conversations. Women should be informed that large scars will be visible in all cases, with particular emphasis on the abdominal scars, as they tend to experience abdominal scars significantly worse than their breast scars [7]. Donor site scars could form a major theme of dissatisfaction and these scars are an aspect of the surgery that participants felt unprepared for, as found in previous studies [8, 11]. We therefore recommend to always ask women how concerned they are about visible scarring of both the breast and abdomen, after which other reconstructive flap sites with scars in a less prominent visible area can also be considered and discussed, if relevant and applicable. Providing specific information on appearance and possible scar-related symptoms could potentially contribute to women having more realistic expectations about scarring and their expected outcomes. Previous literature after breast reconstruction has already demonstrated the impact of sufficient and clear information about possible outcomes, and on having more realistic expectations [11]. This allows patients to make well-informed decisions [27, 28], resulting in more satisfied patients [29].

It is also important to note, that the most widely used and validated instrument exclusively designed to assess satisfaction and HR-QoL, the BREAST-Q, includes an item concerning the appearance of the abdominal scar. However, no items about scarring are included in the other breast scales of BREAST-Q’s reconstructive module. Taking into account the findings of our studies [7, 30], it would be valuable to consider the inclusion of a scar assessment scale alongside the utilization of the BREAST-Q in clinical practice, for a more comprehensive evaluation of patient’s experiences.

## Strengths and Limitations

This is one of the few studies investigating the impact of patient-reported scar symptoms, including both breast and abdominal scars, after DIEP-flap breast reconstruction on patient satisfaction and HR-QoL. The study utilizes commonly used validated measures, providing clinically relevant insights. Nevertheless, we were restricted to available validated scales, and therefore could not explore the abdominal satisfaction for women with no/minor scar symptoms versus more scar symptoms, because no total score could be generated for ‘Satisfaction with abdomen’. The psychosocial well-being related to the abdomen also could not be explored, as this BREAST-Q scale focuses only on the psychosocial well-being related to the breasts. Another limitation of this study is the cross-sectional design, with all limitations inherent to cross-sectional studies. To include a diverse group, women who underwent DIEP-flap breast reconstruction were invited to complete the survey regardless of the presence, severity, or type of scar symptoms, as well as whether they received additional therapy for their scars. Participants represented a wide range of scar maturation stages, and as a result, perceptions of scarring evolved over time [7]. However, based on our sensitivity analyses, it can be concluded that regardless of the time since surgery, there is an observable trend where participants with symptom-related scars also report lower HR-QoL and satisfaction compared to participants experiencing minor or no scar-related symptoms.

Our previous research indicated factors directly associated with a negative scar appraisal, such as a shorter time since surgery, other complaints in the area of the scar, and dissatisfaction with the information provided about the scar [7]. Possible indications for confounding factors were explored, but none were found (see Digital Supplement 1). Due the explorative study design, possible interfering factors were not tested. It is recommended that future prospective studies control for these factors, in addition to other known patient and surgical characteristics affecting satisfaction and HR-QoL after breast reconstruction, to investigate scars as a risk factor for impaired satisfaction and HR-QoL following DIEP-flap breast reconstruction.

Current exploratory research clearly indicates that scars following DIEP-flap breast reconstruction can have a significant impact on satisfaction and HR-QoL. As visible scarring is not specific to DIEP-flap procedures, it is valuable to compare different techniques of breast reconstruction in terms of scar appraisal and their varying impacts on satisfaction and quality of life in future studies. This will enhance our understanding of the impact of such scars, enabling more precise information to be provided during the decision-making process and consideration of different breast reconstruction options.

In conclusion, women experiencing scar symptoms after DIEP-flap breast reconstruction had a clinically relevant and statistically significant lower degree of overall satisfaction and HR-QoL compared to women who had no/minor scar symptoms. We therefore recommend to explicitly and repeatedly address the inevitability of visible scars after DIEP-flap breast reconstruction during consultations and to give sufficient information about scars before and after DIEP-flap breast reconstruction aiming to improve patient selection and expectation management in women with a desire to undergo DIEP-flap breast reconstruction.

## Supplementary Information

Below is the link to the electronic supplementary material.

Supplementary file1 (DOCX 23 KB)

Supplementary file2 (DOCX 17 KB)
