# Supplementary material for: The Impact of Scars After DIEP-Flap Breast Reconstruction on Satisfaction and HR-QoL: A Cross-Sectional Study Comparing BREAST-Q Scores
Source: Aesthetic Plast Surg. 2024 Sep 3;49(3):733–40. doi: 10.1007/s00266-024-04272-y (PMC11870866; doi:10.1007/s00266-024-04272-y)
Supplement: Supplementary file 1 — Supplementary file1 (DOCX 23 KB) [file 266_2024_4272_MOESM1_ESM.docx]

|  | | | BREAST SCAR | | | | ABDOMINAL SCAR | | | |
| --- | --- | --- | --- | --- | --- | --- | --- | --- | --- | --- |
| All patients (n= 248) | | | **No/minor scar complaints**  (n = 128) | | **Major scar complaints**  (n=120) | | **No/minor scar complaints**  (n= 88) | | **Major scar complaints**  (n=160) | |
| N = 248 | **Mean** | **± SD** | **Mean** | **± SD** | **Mean** | **± SD** | **Mean** | **± SD** | **Mean** | **± SD** |
| Age (year) | 51.8 | ± 9.0 | 52.26 | ± 9.5 | 51.4 | ± 8.4 | 53.8 | ± 9.2 | 50.7 | ± 8.7 |
| Time since DIEP | | | | | | | | | | |
| year | 2.6 | ± 1.9 | 2.4 | ± 2.0 | 1.8 | ± 1.7 | 2.5 | ± 2 | 1.9 | ± 1.8 |
| Months | 31.4 | ± 22.3 | 34.6 | ± 23.5 | 27.9 | ± 20.4 | 35.4 | ± 23.5 | 29.2 | ± 21.3 |
| BMI (kg/m^2^) | 27.1 | ± 3.5 | 26.8 | ± 3.4 | 27.4 | ± 3.6 | 27.2 | ± 3.7 | 27 | ± 3.4 |
|  | ***N*** | **%** | ***N*** | **%** | ***N*** | **%** | ***N*** | **%** | ***N*** | **%** |
| Bra cup size |  |  |  |  |  |  |  |  |  |  |
| < D | 150 | 60 | 79 | 62 | 71 | 59 | 58 | 66 | 92 | 58 |
| ≥ D | 98 | 40 | 49 | 38 | 49 | 41 | 30 | 34 | 68 | 42 |
| Time of reconstruction^1^ | | | | | | | | | | |
| Direct | 68 | 28 | 36 | 28 | 32 | 27 | 26 | 30 | 42 | 26 |
| Indirect | 179 | 72 | 91 | 71 | 88 | 73 | 61 | 69 *1mis | 118 | 74 |
| Laterality | | | | | | | | | | |
| Unilateral | 140 | 56 | 76 | 60 | 64 | 53 | 51 | 58 | 89 | 56 |
| Bilateral | 108 | 44 | 52 | 40 | 56 | 47 | 37 | 42 | 71 | 44 |
| History of breast cancer | | | | | | | | | | |
| Breast cancer history | 187 | 75 | 93 | 73 | 26 | 22 | 69 | 78 | 118 | 74 |
| No breast cancer history | 61 | 25 | 35 | 27 | 94 | 78 | 19 | 22 | 42 | 26 |
| Irradiation^2^ | | | | | | | | | | |
| Yes | 99 | 40 | 46 | 36 | 53 | 44 | 34 | 39 | 65 | 41 |
| No | 149 | 60 | 59 | 46 | 47 | 39 | 42 | 48 | 64 | 40 |
| Self-reported complications^3^ | | | | | | | | | | |
| Breast | 105 | 42 | 47 | 37 | 58 | 48 |  |  |  |  |
| Abdomen | 105 | 42 |  |  |  |  | 24 | 27.3 | 81 | 51 |
| Self-reported current symptoms | | | | | | | | | | |
| Breast | 110 | 44 | 47 | 37 | 63 | 53 |  |  |  |  |
| Abdomen | 94 | 38 |  |  |  |  | 19 | 22 | 75 | 47 |
| Note: ^1^ n =247, missing due to incorrect data entry for date.  ^2^43 participants had a bilateral prophylactic mastectomy, no radiation was indicated.^3^ Some of the women (n = 58) had both breast and abdominal complications, however, the number of complications happened to be equally high. | | | | | | | | | | |

**Digital supplement 1: Comparison of Patient Characteristics Based on the Presence or Absence of Scar Complaints**
