# Supplementary material for: The Impact of Scars After DIEP-Flap Breast Reconstruction on Satisfaction and HR-QoL: A Cross-Sectional Study Comparing BREAST-Q Scores
Source: Aesthetic Plast Surg. 2024 Sep 3;49(3):733–40. doi: 10.1007/s00266-024-04272-y (PMC11870866; doi:10.1007/s00266-024-04272-y)
Supplement: Supplementary file 2 — Supplementary file2 (DOCX 17 KB) [file 266_2024_4272_MOESM2_ESM.docx]

**Digital supplement 1: Sensitivity Analyses**

**Sensitivity analysis BREAST-Q scores: scars less than 1 year old vs more than 1 year old**

|  | **Breast-Q scores Scars ≤1 year** | | **Breast-Q scores scars >1 year** | |
| --- | --- | --- | --- | --- |
|  | **no/minor scar symptoms**  POSAS score 1-3 (n) | **(major) scar symptoms**  POSAS score 4-10 (n) | **no/minor scar symptoms**  POSAS score 1-3 (n) | **(major) scar symptoms**  POSAS score 4-10 (n) |
| **BREAST** | | | | |
| **Satisfaction** | 71.8 (15) | 51.5 (28) | 74.8 (113) | 59.0 (92) |
| **Physical well-being** | 82.1 (15) | 59.0 (28) | 79.5 (113) | 67.0 (92) |
| **Psychosocial well-being** | 79.9 (15) | 57.8 (28) | 78.1 (113) | 66.3 (92) |
| **Sexual well-being** | 63.2 (14) | 50.7 (27) | 61.6 (92) | 47.9 (86) |
| **ABDOMINAL** | | | | |
| **Physical well-being** | 66.6 (10) | 52.9 (33) | 70.1 (78) | 57.4 (127) |
| **Sexual well-being** | 66.6 (9) | 51.8 (32) | 61.6 (66) | 51.1 (112) |

**Note.** The POSAS overall opinion was used to divide the participants in no/minor scar symptoms and (major) scar symptoms.

**Sensitivity analysis BREAST-Q scores: scars less than 1,5 year old vs more than 1,5 year old**

|  | **Breast-Q scores scars ≤1.5 year** | | **Breast-Q scores scars >1.5 year** | |
| --- | --- | --- | --- | --- |
|  | **No/minor scar symptoms**  POSAS score 1-3 (n) | **(major) scar symptoms**  POSAS score 4-10 (n) | **No or minor scar symptoms**  POSAS score 1-3 (n) | **(major) scar symptoms**  POSAS score 4-10 (n) |
| **BREAST** | | | | |
| **Satisfaction** | 70.9 (30) | 53.7 (43) | 75.6 (98) | 59.2 (77) |
| **Physical well-being** | 81.2 (30) | 59.6 (43) | 79.4 (98) | 68.2 (77) |
| **Psychosocial well-being** | 73.2 (30) | 60.2 (43) | 79.9 (98) | 66.6 (77) |
| **Sexual well-being** | 61.4 (25) | 51.1 (41) | 62.0 (81) | 47.0 (72) |
| **ABDOMINAL** | | | | |
| **Physical well-being** | 69.2 (17) | 54.5 (56) | 69.8 (71) | 57.6 (104) |
| **Sexual well-being** | 66.6 (14) | 51.8 (52) | 61.1 (61) | 50.9 (92) |

**Note.** The POSAS overall opinion was used to divide the participants in no/minor scar symptoms and (major) scar symptoms.
